# Evaluation of the Therapeutic Potential of Bioactive Materials Based on a Complex of Oxidovanadium(IV) and Exopolysaccharide Levan in a Model of Insulin Resistance in Mice

**DOI:** 10.1002/cmdc.202500754

**Published:** 2025-12-17

**Authors:** Amanda K. J. P. F. da Silva, Eucilene K. de L. B. Marques, Lidiane M. A. de Lima, Widarlane A. S. Alves, Dayane A. Gomes, Pedro L. Guzzo, Mônica F. Belian, Wagner E. Silva, Eduardo C. Lira

**Affiliations:** ^1^ Departamento de Química Universidade Federal Rural de Pernambuco Rua Dom Manoel de Medeiros, S/N—Dois Irmãos Recife‐PE CEP: 52171–900 Brazil; ^2^ Departamento de Fisiologia e Farmacologia Centro de Biociências Universidade Federal de Pernambuco Avenida Professor Moraes Rego, S/N—Cidade Universitária Recife‐PE CEP: 50670–901 Brazil; ^3^ Departamento de Engenharia de Minas Centro de Tecnologia Universidade Federal de Pernambuco Rua Acadêmico Hélio Ramos, S/N—Cidade Universitária Recife‐PE CEP: 50740–530 Brazil

**Keywords:** dexamethasone, diabetes mellitus type 2, insulin resistance, levan matrix, oxidovanadium(IV) complex

## Abstract

Bioactive compositions containing vanadium complexes have been a viable strategy for constructing more biocompatible and less toxic systems. Therefore, this work aim to develop a new composition formed by an oxidovanadium(IV) complex as levan. The acute oral toxicity and insulin resistance (IR) are investigated in an animal model using adult Swiss mice treated with daily injections of the synthetic glucocorticoid dexamethasone. The complex is characterized by electronic absorption (λ_max_ = 771 and 880 nm) and infrared spectroscopies (3359, 3167, 1606, 1342, 1072 cm^−1^, and the V=O at 937 cm^−1^); NMR of the ^1^H, ^13^C, and ^51^V (−427, −509, and −529 ppm), and electron paramagnetic resonance (g‐factor = 1.985). The vanadium complex is classified in category 4, according to the acute toxicity protocol. IR in mice is accompanied by a rise in fasting blood glucose at seventh (2.2‐fold) and 14th (threefold) days, triglyceride levels at seventh (2.6‐fold) and 14th (threefold) days, and triglyceride/glucose index (TyG) at seventh (20%) and 14th (25%) days. The bioactive composition attenuated both the hyperglycemia (≈65%) and hypertriglyceridemia and TyG in a dose‐dependent manner. The proposed composition shows promise in reducing IR induced by exogenous corticosteroid treatment.

## Graphical abstract

A low‐toxicity bioactive composition of oxidovanadium(IV) and exopolysaccharide levan is synthesized, characterized, and shown to reduce hyperglycemia, hypertriglyceridemia, and TyG index in a mice model of dexamethasone‐induced insulin resistance.

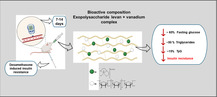

## 1 Introduction

Insulin resistance (IR) is a state characterized by hyperinsulinemia and glucose intolerance, resulting from the reduced responsiveness of insulin‐targeting tissues to physiological insulin levels.^[^ ^1^ ^]^ The main features of IR include decreased lipolysis in adipose tissue, impaired glucose uptake by muscle, impaired/reduced net glycogen synthesis, and suppressed gluconeogenesis in the liver.^[^ ^2^ ^]^ It is well known that IR is a key factor in the development of several metabolic diseases, including Type 2 diabetes mellitus (T2 DM), obesity, nonalcoholic fatty liver disease (NAFLD), cardiovascular disease, and metabolic syndrome (MS). Endocrine disturbances, such as excessive endogenous cortisol levels seen in Cushing's disease and long‐term exposure to excess glucocorticoids (GCs), whether from disease or treatment, gradually lead to the development of IR.^[^ ^3^ ^,^ ^4^ ^]^

GCs are steroid hormones produced by the adrenal cortex under hypothalamic–pituitary–adrenal (HPA) axis regulation, coordinating mammalian glucose homeostasis under both basal and stress conditions.^[^ ^4^ ^]^ In addition, GCs are the most frequently prescribed anti‐inflammatory drugs for treating various diseases, such as rheumatoid arthritis, psoriasis, lupus erythematosus, asthma, and as immunosuppressive agents in organ transplantation rejection regimens.^[^ ^5^ ^]^ However, the full clinical use of these steroids has been restricted because of their undesirable effects. Typically, these side effects are associated with the type of steroid used, the dose, and the duration of treatment.^[^ ^6^ ^]^ It is well established that the clinical use of GCs may contribute to the redistribution of fat deposits, IR, hyperglycemia, and muscle protein catabolism in both rodents and humans.^[^ ^7^, ^8^, ^9^ ^]^

Novel vanadium complexes have emerged as a promising therapeutic approach for mitigating metabolic side effects associated with GC treatment.^[^ ^10^, ^11^, ^12^, ^13^ ^]^ Therefore, existing findings have shown that the use of vanadium complexes can be an alternative to minimizing the side effects of glucocorticoids on glucose, lipid, and protein metabolism in patients with hypercortisolism or those requiring long‐term or high‐dose exogenous glucocorticoid‐based therapy.

**Figure 1:**
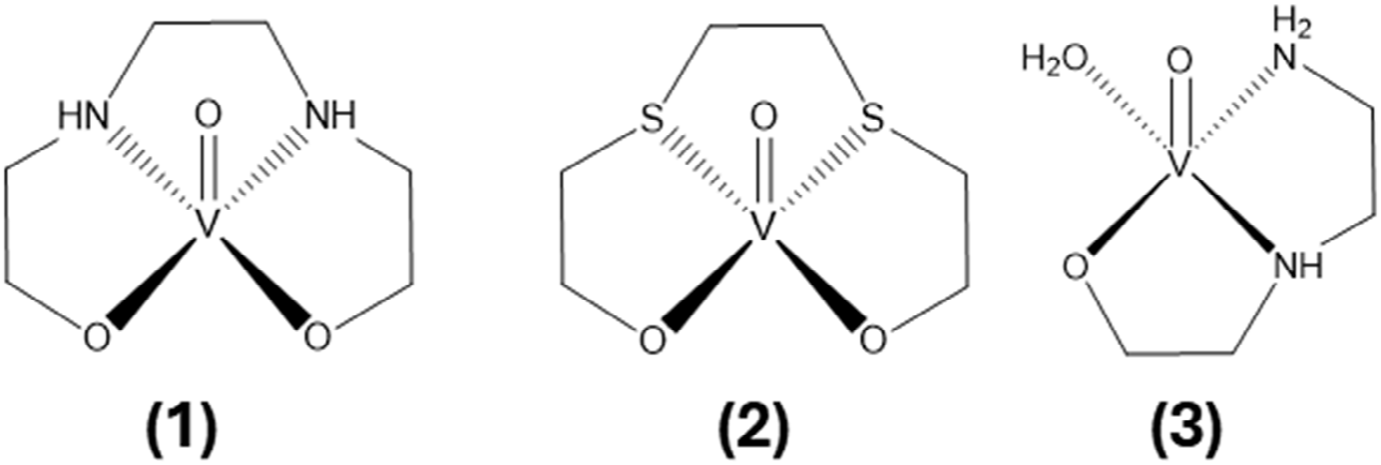
Vanadium compounds structures with aliphatic chelator ligands (**1**) [V^IV^O(BHED)], BHED = *N*‐(2‐bishydroxyethyl)ethylenediamine; (**2**) [V^IV^O(OCDT)], OCDT = 3,6‐dithio‐1,8‐octanediol; and **(3** **)** [V^IV^O(H_2_O)(HEED)]_2_SO_4_, HEED = *N*‐(2‐hydroxyethyl)ethylenediamine*.*

Vanadium complexes (VCs) have been widely studied as potential antidiabetic agents, with evidence from in vitro studies, animal models, and human clinical trials.^[^ ^13^, ^14^, ^15^, ^16^, ^17^ ^]^ Our research group developed two vanadium bioactive complexes coordinated with aliphatic chelators, abbreviated as [V^IV^O(BHED)] (1), BHED = *N*‐(2‐bishydroxyethyl)ethylenediamine,^[^ ^18^ ^]^ and [V^IV^O(OCDT)] (2), OCDT = 3,6‐dithio‐1,8‐octanediol, with antidiabetic properties.^[^ ^19^ ^]^ The [V^IV^O(BHED)] complex promoted a reduction in plasma glucose and improved lipid profile in streptozotocin‐induced diabetic rats in a Type 1 diabetes (T1 DM).^[^ ^18^ ^]^ On the other hand, [V^IV^O(OCDT)] reduced IR and improved oral glucose tolerance in an animal model of dexamethasone‐induced IR in mice (**Figure** **1**).^[^ ^20^ ^]^ Based on these results, which demonstrate that vanadium complexes with aliphatic chelators exhibit insulin‐mimetic activities,^[^ ^21^ ^]^ this manuscript proposes a new vanadium complex using a VO(NO_3_) coordination mode ligand with a structure similar to an ionophore ligand,^[^ ^18^ ^]^ aiming to improve the antihyperglycemic effects.

Polysaccharide matrices have been utilized in bioactive compositions for the treatment of Type 2 Diabetes Mellitus (T2 DM).^[^ ^22^ ^]^ Several studies have described the use of carboxymethyl cellulose, Xanthan gum, and alginic acid in formulations containing vanadium complexes to reduce toxicological effects, enhance biological properties, and increase the solubility of the composition.^[^ ^22^, ^23^, ^24^ ^]^ In this study, a composition combining oxidovanadium complexes with the structural and functional advantages of a bacterial exopolysaccharide was proposed.

Bacterial exopolysaccharides (EPS) are essential natural biopolymers utilized in various fields, including biomedicine, food, cosmetics, petroleum, pharmaceuticals, and environmental remediation. A particular EPS—levan—is a homopolysaccharide composed of monomeric D‐fructofuranosyl units obtained from Gram‐negative bacteria, which has emerged as a promising bioactive agent against diabetes.^[^ ^25^ ^]^ Its nontoxicity, high water solubility, heat stability, and low viscosity make it a safe and reliable candidate for diabetes treatment. Dahech et al. reported that a levan produced by the microorganism Bacillus licheniformis exhibited potential antidiabetic activity in vivo.^[^ ^26^ ^]^ Dahech et al. demonstrated in another study that levan significantly reduced plasma glucose levels and restored oxidative stress (OS) levels, showing its ability to protect liver and pancreatic tissues.^[^ ^27^ ^]^ Furthermore, levan has been shown to reduce total cholesterol, triglycerides, and LDL‐cholesterol levels, further reinforcing its potential in diabetes management.^[^ ^28^ ^]^

This paper reports the development of a composition formed by a novel oxidovanadium complex using the ligand *N*‐(2‐hydroxyethyl)ethylenediamine, abbreviated as [V^IV^O(H_2_O)(HEED)]_2_SO_4_ (3), where HEED = *N*‐(2‐hydroxyethyl)ethylenediaminoate, and levan to reduce toxicity and optimize the glucose‐lowering effects. Toxicity and biological studies of the composition were performed using an insulin resistance (IR) model induced by acute dexamethasone exposure in female mice.

## 2 Experimental Section

### 2.1 Chemicals and General Methods

Oxidovanadium(IV) sulfate hydrate (97%), metallic sodium, and *N*‐(2‐hydroxyethyl)ethylenediamine were purchased from Sigma–Aldrich (St. Louis, MO, USA). Ultrapure water (18.2 mW cm) was used in the synthesis and biological studies with the vanadium‐amino alcohol complex. The NMR data were collected on a Varian Mercury 400 MHz spectrometer (Massachusetts, USA), with frequencies of 78.9 MHz for ^51^V, 300 MHz for ^1^H, and 100 MHz for ^13^C. The ^51^V NMR reference external was VOCl_3_ (0.00 ppm), and TMS was used for ^1^H and ^13^C, with deuterated water (D_2_O) as the solvent.^[^ ^29^ ^]^ Fourier transform infrared (IR) spectra (400 to 4000 cm^–1^) were recorded from ATR mode on the Shimadzu (Tokyo, Japan) spectrophotometer. The EPR spectra were measured using a Bruker EMX 10+ spectrometer operating at X‐band frequencies with a cylindrical cavity and 100 kHz field modulation. The EPR measurements were carried out at room temperature, with the capillary samples placed in 2 mm I.D. vitreous silica tubes of high purity. The measuring parameters were set as follows: (i) microwave power: 0.632 mW; (ii) modulation amplitude: 2 G; (iii) time constant: 81.92 ms; (iv) conversion time: 25 ms; (v) receiver gain: 103; (vi) sweep width: 1100 G; (vii) resolution 2000 points; (viii) number of scans: 8. DPPH was used as a standard to check g‐factor determinations.^[^ ^30^ ^]^ The EPR signals were identified by comparing the g‐factor from V^IV^ and hyperfine splitting values spectra with the parameters reported in the literature.^[^ ^31^ ^]^ An aqueous solution of V^IV^OSO_4_ (50 mmol L^−1^) was used as a control. Electronic absorption spectra of a 10^−3 ^mol L^−1^ aqueous solution of the complex were recorded over a wide range of 300–900 nm using a Shimadzu (Tokyo, Japan) UV–vis–NIR spectrometer. For thermogravimetric analysis (TGA), the Shimadzu DTG‐60H equipment was used under an oxidizing atmosphere (compressed air) with a flow rate of 50 mL min^−1^ and a heating rate of 10 °C min^−1^.

### 2.2 Synthesis of the {*Bis*‐[aquo‐N‐(2‐Hydroxyethyl)ethylenediaminoateoxidovanadium(IV)]}sulfate Complex‐[V^IV^O(HEED)(H_2_O)]_2_SO_4_

In a 100 mL round‐bottom flask, metallic sodium (1 mmol, 0.023 g) was dissolved in 30 mL of methanol (P.A. grade) under stirring at room temperature (25 °C). After the complete dissolution of sodium, *N*‐(2‐hydroxyethyl)ethylenediamine (HEED, 1 mmol, 0.104 g, 101.11 μL, 99%) was added dropwise to the reaction mixture. The solution was stirred at 25 °C for 3 h. Following completion, the solvent was removed under a high vacuum. The resulting white solid was collected by centrifugation and washed with ethanol (3 × 15 mL). The product was dried under vacuum. Yield: 90%

**Scheme 1:**
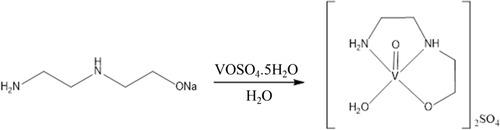
Reaction scheme of [V^IV^O(H_2_O)(HEED)]_2_SO_4_ complex, where HEED = (*N*‐(2‐hydroxyethyl)ethylenediaminoate).

To the sodium *N*‐(2‐hydroxyethyl)ethylenediaminoate sodium salt (1 mmol, 0.126 g), dissolved in 10 mL of methanol, was added VOSO_4_·5H_2_O (1 mmol) under constant stirring at 25 °C (pH reaction ≈7.1). The reaction was allowed to proceed for 24 h (**Scheme** **1**). Afterward, the reaction mixture was frozen at 0 °C and lyophilized to remove water. The resulting green solid was washed with ethanol (3 × 15 mL) and then dried under vacuum. Yield: 85%

Electronic absorption *λ*(_máx_); *ε*(mol^−1 ^L cm^−1^): DMSO = 259 (1650), 330(540), 557, 875 (281), H_2_O = 711, 880 (196); FTIR cm^−1^ (KBr): n 3359 (vOH), 3167 (vNH_2_ and NH), 1606 (vNH), 1342 (vC–N), 1072 (vC–O), and 937 (vV=O); NMR ^1^H (d, ppm, J, D_2_O): 3.28–3.26 (t, J_1–2_ = 3 Hz), 3.47–3.43 (dd, J_1–3_ = 3.0, J_2_ = 6.0 and J_4_ = 12 Hz), and 3.88–3.85 (t, J_1_ = 3.0, J_2_ = 6.0 and J_3_ = 9 Hz); NMR ^13^C (d, ppm, D_2_O): 35.41, 49.55, 56.45, and 76.25.

### 2.3 Production and Purification of Levan

The *Zymomonas mobilis* strain, registered in the Collection of Microorganisms of the Department of Antibiotics—Universidade Federal de Pernambuco, UFPEDA 241, was used in this work.^[^ ^32^ ^]^ The collection is registered with the World Federation of Culture Collections—World Data Center on Microorganisms (number 114). It was cultivated in Standard Swings & De Ley (SDL) medium, containing 20.0 g L^−1^ sucrose and 5.0 g L^−1^ yeast extract, at pH 6.5, under refrigeration at −4 °C.^[^ ^33^ ^]^

The production of bacterial exopolysaccharide levan from *Zymomonas mobilis*, which involves the steps of inoculum preparation, prefermentation, and fermentation medium preparation, was performed according to Calazans (2000).^[^ ^34^ ^]^ Exopolysaccharide was precipitated with ethanol, recovering all levan fractions.^[^ ^35^ ^]^ After 24 h, the resulting precipitate was centrifuged, transferred to Falcon tubes, frozen for 12 h at −80 °C, and subsequently lyophilized for 48 h at −50 °C under high vacuum.

### 2.4 Composition Synthesis of the Oxidovanadium Complex and Levan

The novel composition was prepared using an oxidovanadium complex and a levan matrix in a 95:5 (m/m) ratio. This dose was chosen based on Levan's antidiabetic properties, as described by Dahech (2011), who used a 5% (m/m) concentration.^[^ ^26^ ^]^ The vanadium complex and levan were dissolved in a 0.9% saline solution (NaCl) and left under magnetic stirring at room temperature for 24 h.

The tests were performed with the conjugate at 25 mg kg^−1^ (V_25_ + Lev) and 50 mg kg^−1^ (V_50_ + Lev), where V = [V^IV^O(H_2_O)(HEED)]_2_SO_4_.

### 2.5 Animals and Ethical Statement

Female Swiss mice (35 ± 3 g body weight) were maintained in sanitized polypropylene cages (3 per cage for acute toxicity test and 5 per cage for IR model) under standard conditions of temperature (23 ± 2 °C), relative humidity (55 ± 5%), and a 12 h light/12 h dark photoperiod, with ad libitum access to food and water. All animal care procedures were carried out according to the National Institutes of Health Guide for the Care and Use of Laboratory Animals (NIH Publication number 8023) and were approved by the Ethics Committee on the Use of Animals of the Federal University of Pernambuco (CEUA/UFPE, protocol number 0061/2022.)

### 2.6 Acute Oral Toxicity Protocol

The acute toxicity test was conducted in accordance with Guideline 423 (Acute Toxic Class Method) of the Organization for Economic Cooperation and Development (OECD) for assessing the acute oral toxicity of chemicals.^[^ ^36^ ^]^ After 5 days of acclimation in a propylene cage, nulliparous and nonpregnant Swiss female mice (3 for each group) were fasted for 3 h and weighed before vanadium compound administration. The [V^IV^O(H_2_O)(HEED)]_2_SO_4_ was dissolved in NaCl 0.9% (m/v) and administered by gavage in mice in single doses using animal feeding needles (100 μL/100 g b.w.). Animals were randomly divided into three groups with three animals each: (a) treated with NaCl 0.9% (m/v) (**Control**); (b) treated with 5 mg kg^−1^ of [V^IV^O(H_2_O)(HEED)]_2_SO_4_ (**V**_**5**_); (c) treated with 50 mg kg^−1^ of [V^IV^O(H_2_O)(HEED)]_2_SO_4_ (**V**_**50**_); (d) treated with 300 mg kg^−1^ of [V^IV^O(H_2_O)(HEED)]_2_SO_4_ (**V**_**300**_); and (e) treated with 2,000 mg kg^−1^ of [V^IV^O(H_2_O)(HEED)]_2_SO_4_ (**V**_**2000**_).

In the first 4 h, all animals were closely observed for piloerection, changes in skin, fur, and eyes, toxic effects on the mucous membrane, behavior pattern disorientation, hypoactivity, hyperventilation, asthenia, lethargy, sleep, diarrhea, tremors, salivation, convulsion, coma, motor activity, or death. After this, the animals were observed daily for 14 days. Weight gain, food, and water intake were monitored daily. On the 14th day, female mice were euthanized by injection of xylazine (150 mg kg^−1^, i.p.) and ketamine (20 mg kg^−1^, i.p.) solution, and blood and organs were collected for biochemical and macroscopic analysis. Based on mortality in each group, the toxicological potential of the tested samples and the LD_50_ was estimated according to the score: nontoxic samples (zero deaths at a dose of 2000 mg kg^−1^), moderate toxicity (between zero and one death at doses from 300 to 2000 mg kg^−1^), high toxicity (between one or more deaths at doses from 5 to 300 mg kg^−1^).^[^ ^37^ ^,^ ^38^ ^]^

### 2.7 Insulin Resistance Induction

**Figure 2:**
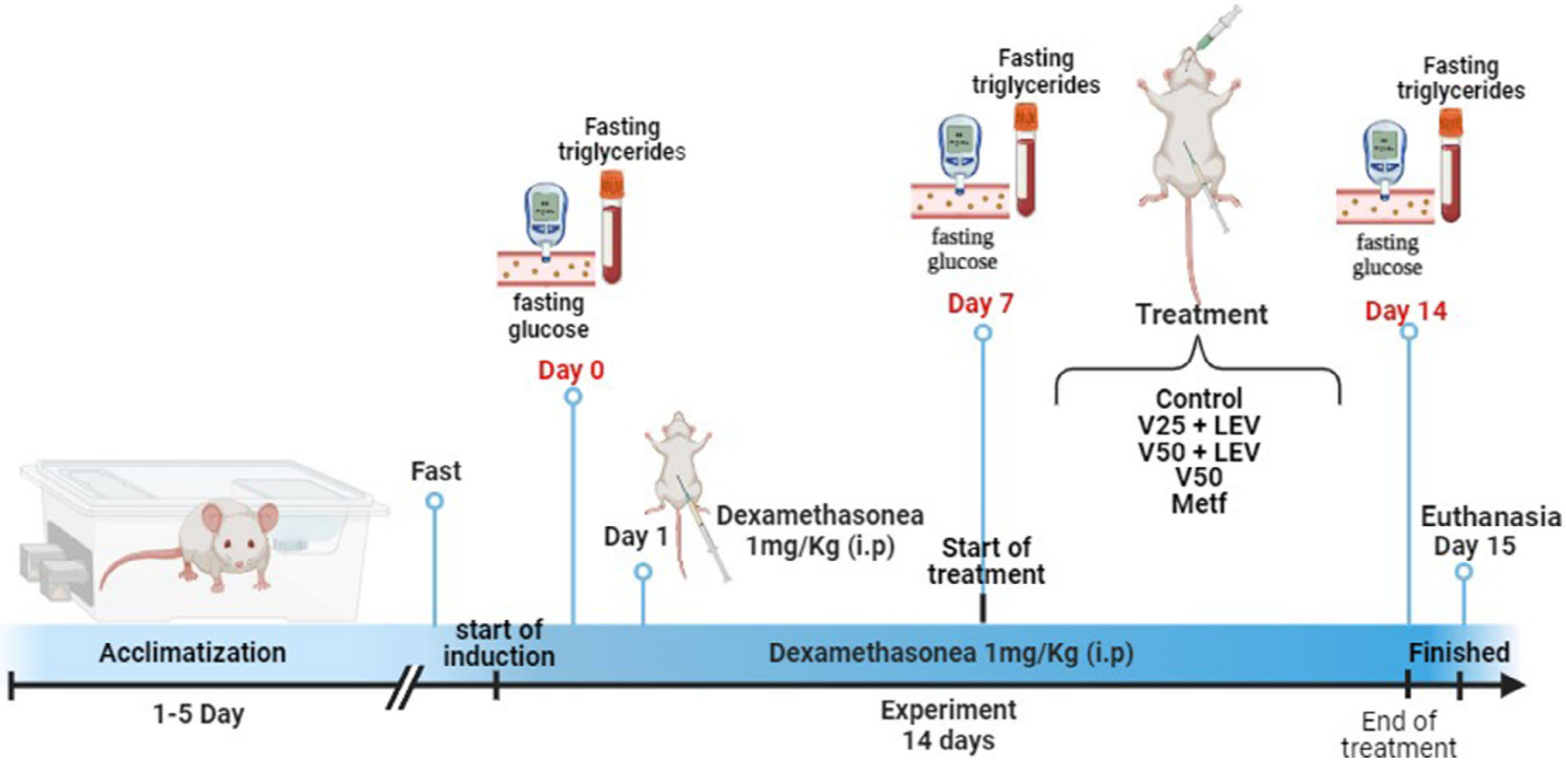
Illustrative scheme of insulin resistance induction protocol using dexamethasone in mice (created with BioRender).

Insulin resistance was induced in female mice (*n* = 5 per group) by intraperitoneal injection of dexamethasone (Aché, Brazil) administration (DEXA, i.p., 1 mg kg^−1^ body weight) for 7 consecutive days (**Figure** **2**), as reported previously by Batista (2024).^[^ ^20^ ^]^ The animals were divided into 6 groups (*n* = 5) abbreviated as (a) control group (**Control**); which received only NaCl 0.9% m/v during 14 days of treatment (1 mL kg^−1^, v.o.), (b) dexamethasone control group (**Dexa**), the animal has only administrated dexamethasone for next 7 days; (c) 25 mg kg^−1^ of [V^IV^(HEED)(H_2_O)]·3H_2_O in levan 5% (m/m) (**V**_**25**_ **+Lev**); (d) (50 mg kg^−1^ of [V^IV^(HEED)(H_2_O)]·3H_2_O in levan 5% (m/m) (**V**_**50**_ **+Lev**); (e) 50 mg kg^−1^ of [V^IV^(HEED)(H_2_O)]·3H_2_O (**V**_**50**_); and (f) metformin (200 mg kg^−1^, i.p. 7 days) (**Met**). Tested groups **Met**, **V**_**25**_ **+Lev, V**_**50**_ **+Lev,** and **V**_**50**_ received oral administration of compounds combined intraperitoneal administration of dexamethasone for 7 days.

### 2.8 Biochemical Analysis

On the 14th day after treatment (toxicity and insulin resistance protocol), animals were anesthetized and blood was collected and then was centrifuged at 3000 rpm for 15 min at room temperature and serum was separated for the evaluation biochemical parameters.

In acute toxicity test, the serum levels of alanine (ALT) and aspartate aminotransferase (AST), blood urea nitrogen (BUN), creatinine (CRE), total cholesterol (TC), total protein (TP), triacylglycerol (TG), and blood glucose (GLU) were measured by colorimetric assay using commercial kits (Lab Test Diagnostic SA, Santa Lagoa, Brazil). The non–C–HDL levels were calculated as (TC) – (C‐HDL).^[^ ^39^ ^]^

On the 7th and 14th days in the IR model, fasting triacylglycerol and glucose levels were measured to calculate the TyG index (Equation (1)).^[^ ^40^ ^]^

(1) $${TyG}_{index}=\frac{ln\left[TG\left(\frac{mg}{dL}\right)\cdot GLU\left(\frac{mg}{dL}\right)\right]}{2}$$

Optical densities were measured using a spectrophotometer (Varioskan TM Lux multimode microplate reader, Thermo Scientific, Waltham, MA, USA) at wavelengths specific to each biochemical parameter, as described in the datasheets. Baseline measurements were obtained by comparing the optical densities of the samples with those of the respective standards provided in the kits. Data were expressed by U/mL (AST and ALT) and mg/dL for the others.

### 2.9 Statistical Analysis

The data were expressed as mean ± SEM (standard error of the mean). A one‐way analysis of variance (ANOVA) followed by the Bonferroni test was employed to analyze the data, comparing the treated groups with their respective control groups. *P* value < 0.05 was considered statistically significant. The statistical analysis was performed using GraphPad Prism, version 9.4.1.

## 3 Results and Discussion

### 3.1 Chemical Characterization of Bacterial Exopolysaccharide Levan

The production of the exopolysaccharide levan from the bacterium *Zymomonas mobilis*, as reported in a previous study, was successfully carried out.^[^ ^32^ ^]^ The total fraction was obtained by adding ethanol to the medium containing the total fraction, resulting in a crystalline white solid. The total fraction was used because it previously demonstrated antidiabetic properties in vivo.^[^ ^26^ ^,^ ^27^ ^,^ ^41^ ^]^

**Figure 3:**
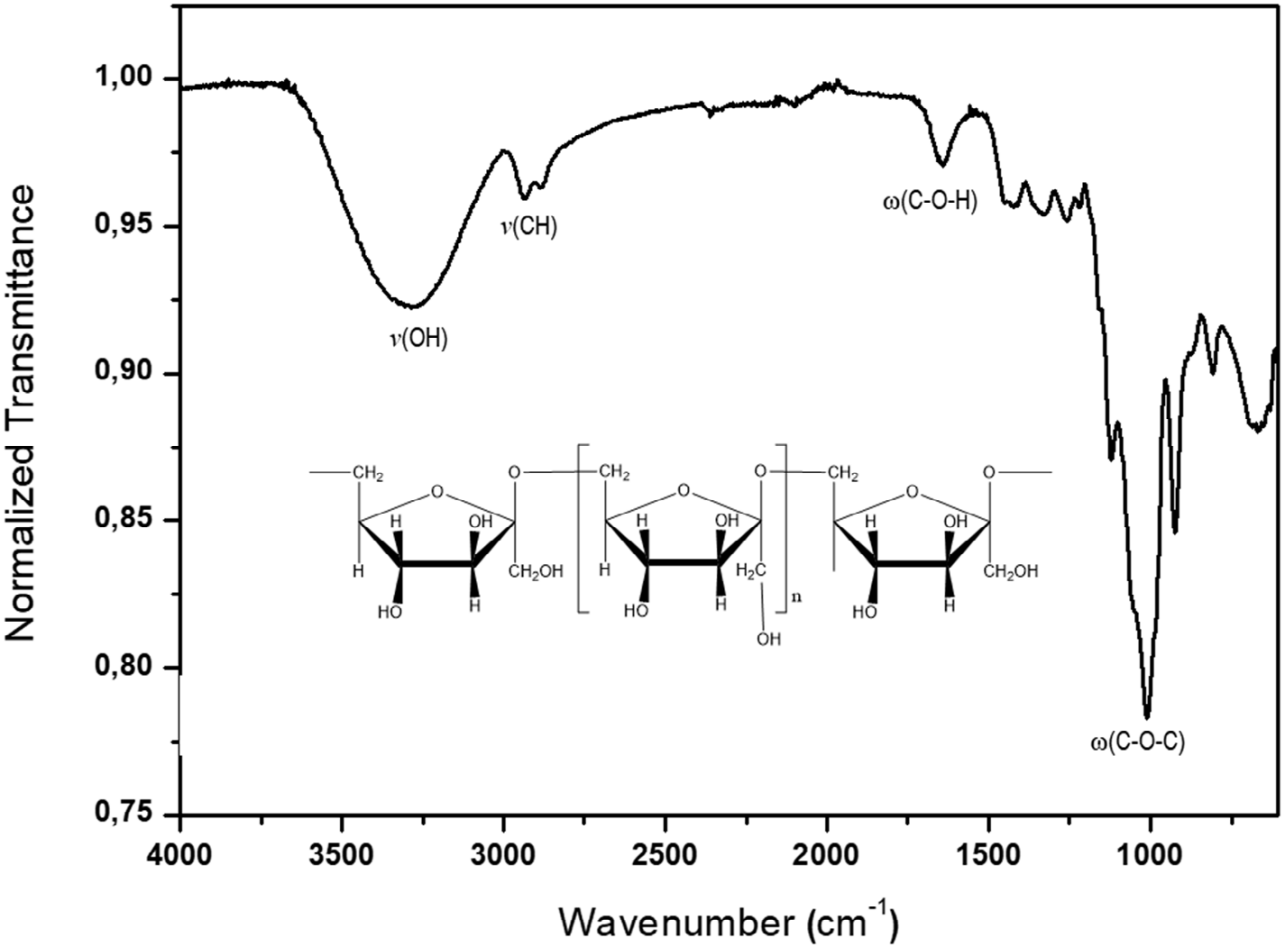
FTIR spectrum of bacterial exopolysaccharide (levan) produced by *Zymomonas mobilis.*

The FTIR spectrum of levan (**Figure** **3**) exhibits a signal at 3302 cm^−1^, attributed to the stretching of the OH group, characteristic of hydrogen bonding. At 2937 and 1425 cm^−1^, the stretching of the C—H and C—O—H bonds is observed, respectively, and the region between 1125 and 917 cm^−1^ refers to the C—O—C bonds from the furanosidic ring.^[^ ^42^ ^]^

The nuclear magnetic resonance (NMR) ^1^H signals are consistent with the literature,^[^ ^34^ ^]^ confirming the formation of bacterial exopolysaccharide (levan) (Figure S1 and S2, Supporting Information).

### 3.2 Chemical Characterization of {*Bis*‐[aquo‐N‐(2‐Hydroxyethyl)ethylenediaminoateoxidovanadium(IV)]}Sulfate Complex‐[V^IV^O(HEED)(H_2_O)]_2_SO_4_

The [V^IV^O(H_2_O)(HEED)]_2_SO_4_ complex, where HEED = (*N‐*(2‐hydroxyethyl)ethylenediamine) as shown in Scheme 1. The resulting dark green solid oxidovanadium(IV) complex has the ligand coordinated through NO_3_ functionalities. The ^1^H, ^13^C, and ^51^V NMR were used to determine the structure of the complex. The ^1^H NMR ligand signals (400 MHz, δ ppm, D_2_O): 3.64 (d, *J* = 6.0 Hz, 1H), 2.69 (d, *J* = 57.0 Hz, 3H), 2.66–2.58 (m, 2H) were shifted in comparison to the ^1^H NMR complex signals (400 MHz, δ ppm, D_2_O): 3.28–3.26 (t, J_1‐2_ = 3 Hz); 3.47–3.43 (dd, J_1–3_ = 3, J_2_ = 6, e J_4_ = 12 Hz); 3.88–3.85 (t, J_1_ = 3, J_2_ = 6, e J_3_ = 9 Hz). Also, the NMR ^13^C ligand signals (d, δ ppm, D_2_O) δ 39.96, 49.97, 50.60, and 60.17 were shifted to ^13^C NMR complex signals (d, δ ppm, D_2_O): 35.41; 49.55; 56.45 and 76.25, (Figure S3 A–B and S4A–B, Supporting Information, respectively) confirms the ligand coordination to the VO^2+^ center.

**Figure 4:**
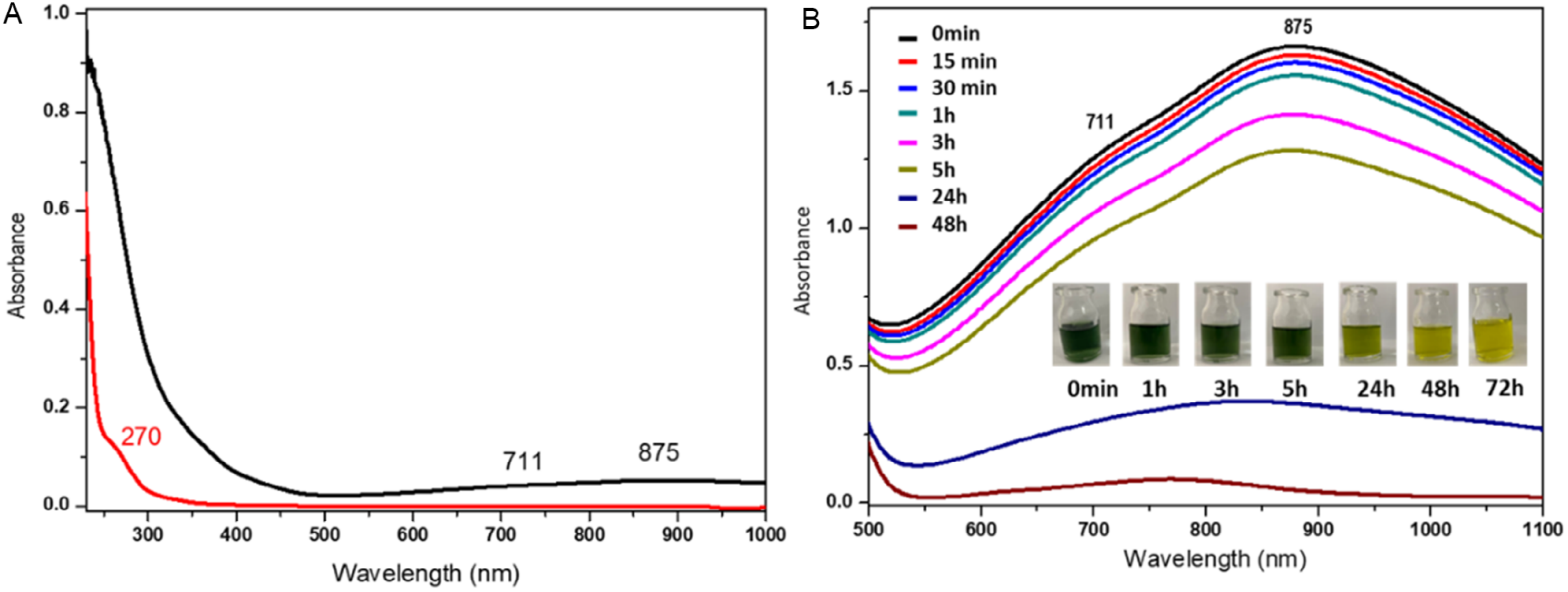
A) Electronic absorption spectra of the [V^IV^O(H_2_O)(HEED)]_2_SO_4_ complex (black line) and HEED (red line) in aqueous solution; B) [V^IV^O(H_2_O)(HEED)]_2_SO_4_ complex at different times (from 0 to 48 h), showing the oxidized species of the [V^IV^O_2_(H_2_O)(HEED)]_2_SO_4_ complex.

The electronic absorption spectra of the ligand (HEED) (**Figure** **4**a, red line) show a band with a maximum at 270 nm (*ε*270 = 440 mol^−1 ^L cm^−1^) characteristic of the n → *σ*∗ transition, referring to nonligand pairs of electrons of the N and O atoms present in the structure of the *N‐*(2‐hydroxyethyl)ethylenediamine. On the other hand, the electronic absorption spectrum of the [V^IV^O(H_2_O)(HEED)]_2_SO_4_ complex (Figure 4a, black line) shows one band at 875 nm (*ε*_257_ = 610 mol^−1 ^L cm^−1^), assigned to V^IV^, d → d intervalence charge transfer transition (IVCT) characteristic of the mixed valence systems of V^IV^ and V^V^.^[^ ^43^ ^]^

Time‐dependent studies were performed to investigate decomposition (or speciation) of the complex solution, using electronic absorption (UV–vis) spectroscopy, ^51^V NMR, and EPR.^[^ ^44^, ^45^, ^46^ ^]^ The UV–vis data support the hypothesis of V^IV^ species oxidation from time zero (at the moment of dissolution) to 48 h (Figure 4b). The decrease in absorbance suggests that after 15 minutes, the V^IV^ complex–[V^IV^O(H_2_O)(HEED)]_2_SO_4_, characterized in the green solution, is oxidized to the V^V^ complex–[V^IV^O(H_2_O)(HEED)]_2_SO_4_ over time.^[^ ^18^ ^,^ ^19^ ^]^ After 24 h, all the V^IV^ species are oxidized to the V^V^ complex, characteristic of the yellow aqueous solution.

**Figure 5:**
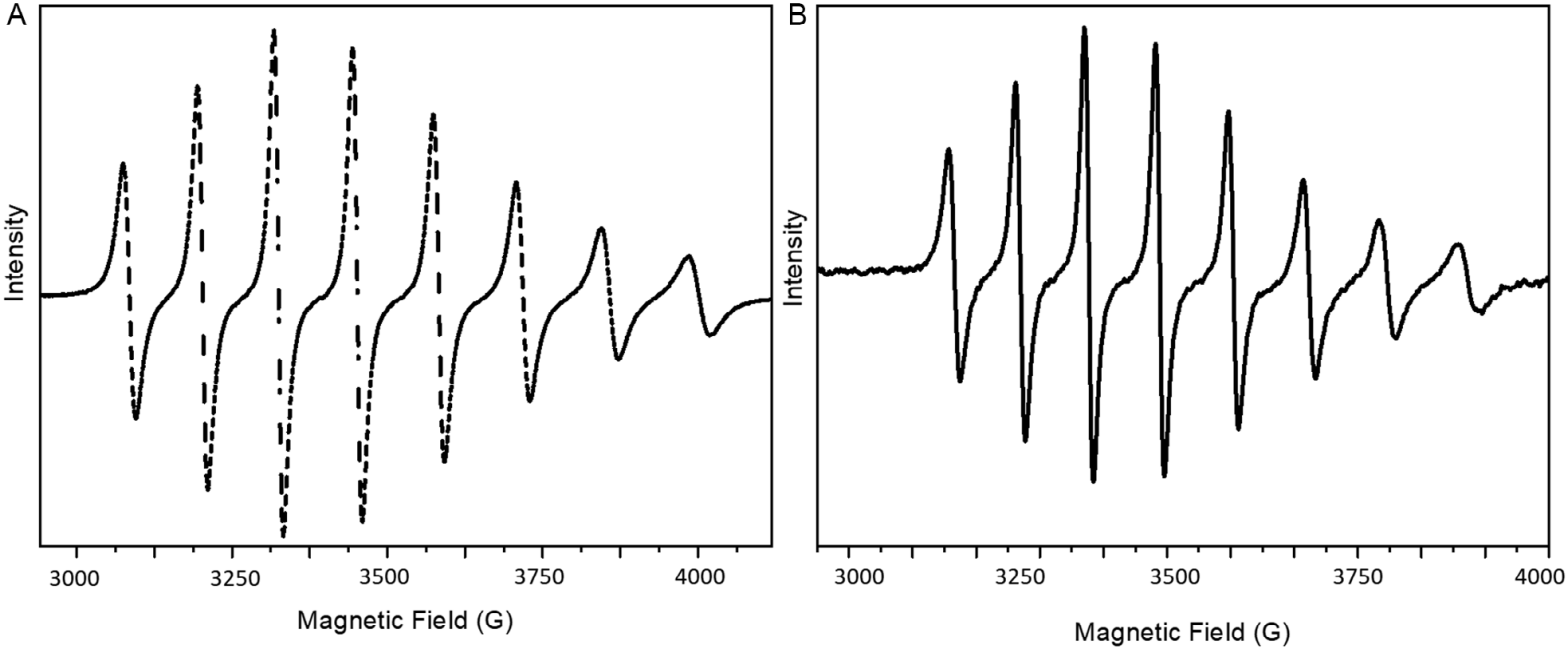
A) The ambient room temperature EPR spectra of 10 mmol vanadyl sulfate and B) [V^IV^O(H_2_O)(HEED)]_2_SO_4_ complex, both in aqueous solution (*t* = 0 h).

The ambient room‐temperature EPR spectra for vanadyl sulfate (**Figure** **5**a) and the vanadium(IV) complex (Figure 5b) upon dissolution (*t* = 0 h) showed an eight‐line pattern (N = 2*I* + 1). Once the nuclear spin moment for vanadium paramagnetic V^IV^ (^51^V) is *I* = 7/2, consistent with the vanadium nuclear hyperfine couplings.^[^ ^47^ ^]^ The calculated g‐factor values for the VOSO_4_ and complex are 1.985 and 2.014, consistent with tabulated values for paramagnetic V^IV^ species,^[^ ^30^ ^]^ confirming the formation of the proposed complex shown in Scheme 1.

According to the ^51^V NMR spectrum in aqueous solution (Figure S5, Supporting Information), no signal was observed immediately after solubilization (*t* = 0 h), characteristic of a V^IV^ compound with paramagnetic properties. After 5 h, V_1_ = H_2_VO_4_^−^ (−529 ppm) and V_10_ = V_10_O_28_^6−^ (−509 and −529 ppm) signals are observed to form from the oligomerization of vanadate at pH 4.^[^ ^48^ ^,^ ^49^ ^]^ The ^51^V NMR spectrum after 24 h (in blue) shows that the V_10_ signals increase as time increases, and 3 signals of V_10_ = V_10_O_28_^6−^ species were observed (−427, −509, and −529 ppm).^[^ ^49^ ^]^ The literature confirms that decavanadates are the most stable species in the pH range of 4–6.^[^ ^19^ ^,^ ^29^ ^]^ NMR spectroscopy was used as a sensitive tool for characterizing the electronic properties of vanadium(V) systems. Since vanadium complexes generally have ^51^V NMR chemical shifts in the range of −300 to −700 ppm, in this work, we investigated which V species are responsible for the biological effects.^[^ ^50^ ^]^ The V^IV^ complex remains stable immediately upon dissolution in water, oxidizing to V^V^ over time due to the increased concentration of oxygen species dissolved in the water, as supported by V_1_ = H_2_VO_4_^−^ (−529 ppm) signal in ^51^V NMR (*t* = 1 h) and a decrease in absorbance in electronic absorption spectroscopy after 15 min. In addition, the signal absence in the ^51^V NMR (*t* = 0 h) associated with the 8‐hyperfine lines in the EPR spectrum recorded immediately upon dissolution confirms that an oxidovanadium(IV) coordination compound [V^IV^(HEED)(H_2_O)]_2_SO_4_·5H_2_O, was successfully obtained with high stability in aqueous solution under the conditions required for biological experiments.

The infrared spectrum (FTIR) of the complex (Figure S6, Supporting Information data) shows bands at 3359, 3167, 1606, 1342, and 1062 cm^−1^, corresponding to the OH, NH2, NH, CN, and CO stretching vibrations, respectively. Furthermore, the band observed at 937 cm^−1^ is assigned to the V=O (vanadyl) stretching, consistent with previous reports for oxidovanadium(IV) complexes.^[^ ^51^ ^]^ The position of the carbon–oxygen stretching band (*ν*C—O) was shifted from 1066 to 1071 cm^−1^, and carbon–nitrogen *ν*C—N was shifted from 1386 to 1342 cm^−1^, respectively, compared to the free ligand *N‐*(2‐hydroxyethyl)ethylenediamine, which confirms that NO_3_ is the coordination mode of the ligand. A summary of the principal FTIR bands assignments of the HEED (free ligand), VOSO_4_, and vanadium complex [V^IV^O(H_2_O)(HEED)]_2_SO_4_ is described in **Table** **1**.

**Table 1:** Principal band assignment for the FTIR spectra of the HEED (free ligand), VOSO_4_, and [V^IV^O(H_2_O)(HEED)]_2_SO_4_ complex, respectively.

Wavenumbers [cm^−1^]			
Assignment	HEED	VOSO_4_	[V^IV^O(H_2_O)(HEED)]_2_SO_4_
vOH	3379	3033	3359
vNH_2_ e NH	3265	–	3167
vNH	1649	–	1606
*ω*NH	1422	–	1451
vC—N	1386	–	1342
vC—O	1066	–	1072
vV=O	–	965	937

**Figure 6:**
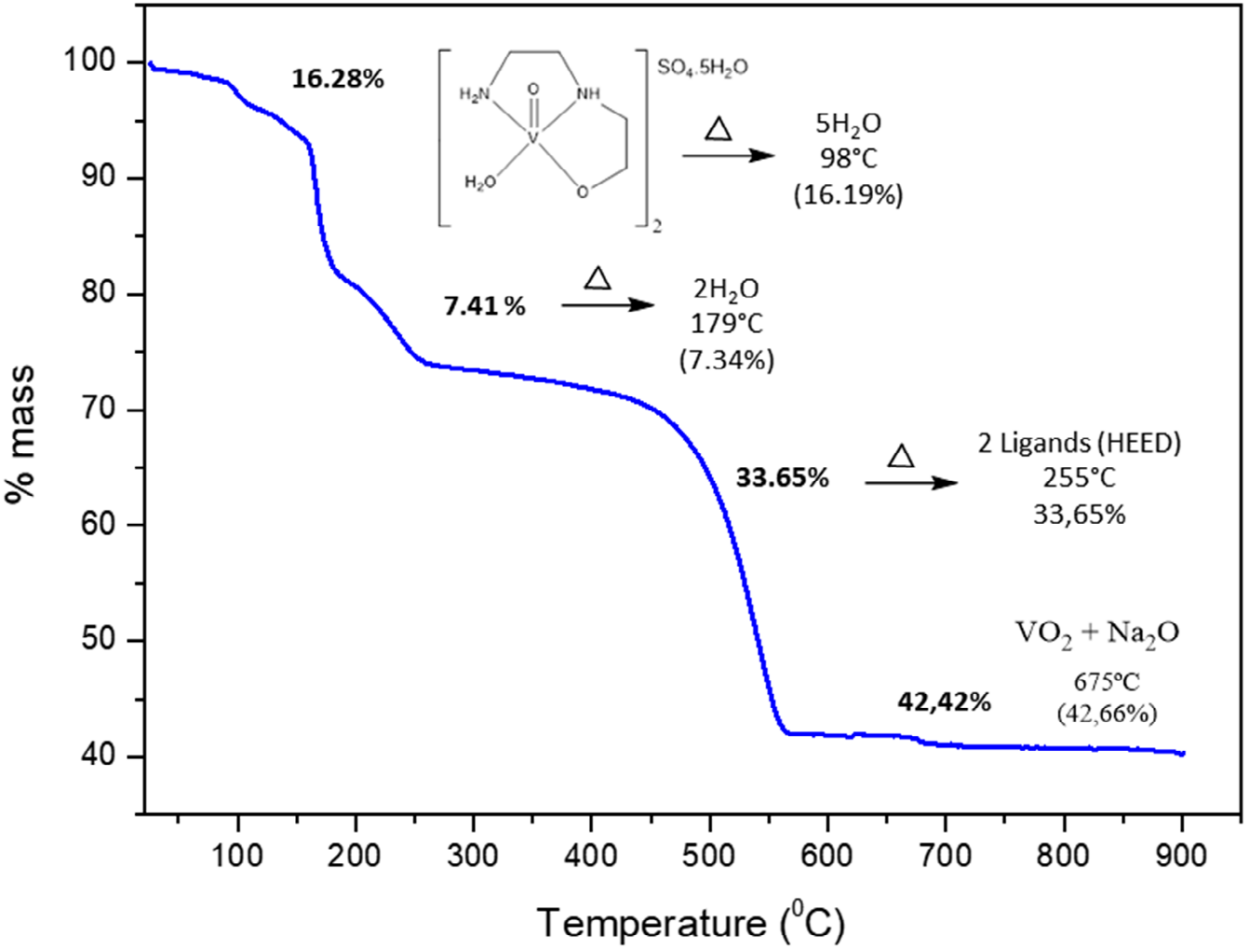
The thermogravimetric curve of the [VO(C_4_N_2_O_2_H_13_)]_2_SO_4_·5H_2_O complex (synthetic air).

**Figure** **6** displays the thermogravimetric analysis (TGA) spectrum of the vanadium complex, which shows three weight‐loss events. The first thermal event at 98 °C (16.28% theoretical weight loss; 16.19% calculated) was attributed to the five hydration water molecules in the second coordination sphere. The second thermal event at 179 °C corresponds to the loss of two coordination water molecules (7.41% theoretical weight loss; 7.34% calculated). The third thermal event at 255 °C (33.65% weight loss theoretical; 34.05% calculated) is related to the decomposition of two ligands, *N*‐(2‐hydroxyethyl)ethylenediamine, from the coordination sphere. The analysis leaves a residue corresponding to 42.42% by mass, attributed to the formation of vanadium oxide (VO_2_) and sodium oxide (Na_2_O), the latter arising from residues of the reference.^[^ ^43^ ^]^ Thermogravimetric analysis indicates that the compound has a minimum formula of [VO(C_4_N_2_O_2_H_13_)]_2_SO_4_·5H_2_O.

### 3.3 Acute Toxicity

Animal studies have shown that the toxicological effects of vanadium compounds are related to the vanadium species (ion), the nature of the complex, the route of administration, and, especially, the dose.^[^ ^52^, ^53^, ^54^, ^55^ ^]^ Several papers describe coordination compounds as less toxic than simple vanadium salts because the ligands enhance the complex's stability in a physiological environment, helping to reduce harmful effects in vitro and in vivo.^[^ ^55^ ^,^ ^56^ ^]^

At a higher dose of 2000 mg kg^−1^, the compound caused a 50% mortality rate. As a result, the [V^IV^O(H_2_O)(HEED)]_2_SO_4_ complex was classified in category 4 (moderate toxicity) according to the OECD 423 protocol (LD_50_ value of 300–2000 mg kg^−1^). Food and water intake, as well as body mass and body weight gain, were not affected by a single oral vanadium complex dose. As shown in **Table** **2**, minor differences were observed, and the food intake increased by 4% (*P* < 0.05) in the V_50_ and V_300_ treated mice at 50 and 300 mg kg^−1^ groups.

**Table 2:** Effects of the acute oral administration of [V^IV^O(H_2_O)(HEED)]_2_SO_4_ on total body weight (g), food intake (g), and fluid intake (mL) during 14 days in different female mice groups.

Parameters	Control	**V**_**5**_	**V**_**50**_	**V**_**300**_
Total body weight (g)	462.0 ± 9.6	455.0 ± 13.0	445.0 ± 10.8	448.5 ± 29.6a)
Food intake (g)	90.0 ± 6.0	82.0 ± 8.0	86.0 ± 6.0	91.0 ± 11.0
Fluid intake (g)	222.0 ± 27.0	204.0 ± 23.0	204.0 ± 17.0	225.0 ± 23.0

The data are presented as the mean ± SEM (*n* = 3). Values are statistically significant at

a) *p* < 0.05 compared to the control group.

The biochemical analysis results are shown in **Table** **3**. The [V^IV^O(H_2_O)(HEED)]_2_SO_4_ oral treatment caused a decrease of the aspartate aminotransferase (AST) at 35% (*P* < 0.05) in the 5 mg kg^−1^ group, at 2.5‐fold (*P* < 0.05) and 1.5‐fold (*P* < 0.05) in the 50 and 300 mg kg^−1^ groups. The alanine aminotransferase (ALT) levels decreased by 8% (*P* < 0.05) for the V_300_ groups compared with untreated mice. Conversely, the vanadium complex did not demonstrate any alteration in blood urea nitrogen (BUN) and total protein levels. Globulin, total proteins, and albumin showed no statistical difference between the groups. These results suggest that the vanadium complex did not demonstrate toxicity at the studied doses in mice.

**Table 3:** Effects of acute oral administration of [V^IV^O(H_2_O)(HEED)]_2_SO_4_ in different mice groups on AST, ALT, albumin, creatinine, BUN, globulin, and total proteins.

Biochemical parameter	Control	**V**_**5**_	**V**_**50**_	**V**_**300**_
AST (U/L)	56.2 ± 6.0	40.9 ± 12.1	22.3 ± 18.6a)	36.4 ± 5.0a)
ALT (U/L)	114.5 ± 8.0	122.2 ± 11.7	105.3 ± 18.8	104.7 ± 8.0a)
Albumin (g L^−1^)	1.9 ± 0.1	2.1 ± 0.1	2.4 ± 0.2	1.9 ± 0.0
Total proteins (g L^−1^)	5.3 ± 0.2	4.8 ± 0.3b)	5.3 ± 0.2	5.3 ± 0.2
Globulin (g L^−1^)	3.4 ± 0.2	2.3 ± 0.4	2.8 ± 0.2	3.2 ± 0.2
BUN (mg L^−1^)	50.4 ± 3.6	59.8 ± 1.9	55.4 ± 4.8	53.0 ± 4.6

The data are presented as the mean ± SD (*n* = 3). Values are statistically significant at

a) *p* < 0.05 compared to the control group and;

b) *p* < 0.05 V_300_ group compared to V_5_ (5 mg kg^−1^ [V^IV^O(H_2_O)(HEED)]_2_SO_4_), V_50_ (50 mg kg^−1^ [V^IV^O(H_2_O)(HEED)]_2_SO_4_) using one–way ANOVA.

This study evaluated the oral acute toxicity and insulin‐enhancing activity of the novel oxidovanadium compound—[V^IV^O(H_2_O)(HEED)]_2_SO_4_—in vivo. The compound exhibited a significant reduction in toxicity compared to previously reported vanadium compounds with antidiabetic activity in the literature.^[^ ^37^ ^,^ ^57^, ^58^, ^59^ ^]^ For example, the NaVO_3_, VOSO_4_·5H_2_O,^[^ ^57^ ^]^ vanadium–rutin complex,^[^ ^58^ ^]^ and vanadium–catecholate complex^[^ ^59^ ^]^ showed LD_50_ values in mice of 74.6, 467.2, 120.0, and 300–2000 mg kg^−1^, respectively, after 14‐day acute oral toxicity administration. In this study, the reported LD_50_ for acute toxicity of [V^IV^O(H_2_O)(HEED)]_2_SO_4_ is 2000 mg kg^−1^. Additionally, the compound demonstrated no signs of nephrotoxicity or hepatotoxicity in the tested dosing mice and effectively reduced the side effects of dexamethasone on carbohydrate metabolism in the mice. These studies are essential because some vanadium coordination compounds have undesirable side effects in animals and humans. Evaluating the toxicological profile of a new drug is the first step in determining any potential risk to human health.^[^ ^37^ ^]^

### 3.4 Dexamethasone–Induced Insulin Resistance in Mice

On the 7th day, fasting plasma blood glucose (FPG, ≈2.1‐fold, *p* < 0.05), triglycerides (fTG, ≈2.6‐fold, *p* < 0.05), and the TyG index (20%, *p* < 0.05) increased in all groups treated with dexamethasone when compared to the control group (*P* < 0.05). In addition, FPG and fTG (≈threefold, *P <* 0.05) and the TyG index (30%, *p* < 0.05) remained elevated during the 14‐day treatment period compared to the control group, confirming that insulin resistance had been induced by glucocorticoids in the clinical condition (**Table** **4**).^[^ ^20^ ^]^

**Table 4:** Effects of fasting plasma blood glucose (FPG), fasting triglycerides (fTG) and tyG index in the control normoglycemic, dexa (1 mg kg^−1^), [V^IV^O(H_2_O)(HEED)]_2_SO_4_ 25 mg kg^−1^ + LEV (V_25_ + lev), [V^IV^O(H_2_O)(HEED)]_2_SO_4_ 50 mg kg^−1^ + LEV (V_50_ + lev), [V^IV^O(H_2_O)(HEED)]_2_SO_4_ 50 mg kg^−1^ (V_50_) and metformin groups on days 0, 7 and 14 of dexamethasone‐induced insulin resistance treatment.

Day	Control			Dexa		
	FPG	fTG	TyG	FPG	fTG	TyG
0	74.80 ± 3.30	55.40 ± 1.90	7.60 ± 0.04	65.40 ± 1.70	67.30 ± 3.90	7.70 ± 0.10
7	71.20 ± 3.50	66.20 ± 3.60	7.80 ± 0.09	158.10 ± 11.10a)	172.30 ± 13.30a)	9.50 ± 0.10a)
14	70.00 ± 3.70	70.10 ± 4.20	7.70 ± 0.05	196.70 ± 8.80a)	208.40 ± 11.60a)	9.90 ± 0.10a)
Day	V_50_			V_25_ + Lev		
0	63.00 ± 5.60	73.40 ± 4.50	7.50 ± 0.10	55.20 ± 2.60	56.30 ± 4.00	7.30 ± 0.10
7	159.20 ± 0.20a)	176.80 ± 7.60a)	9.50 ± 0.10a)	164.60 ± 8.40a)	176.40 ± 5.90a)	9.60 ± 0.10a)
14	70.00 ± 3.50a), b)	108.20 ± 5.60a), b)	8.40 ± 0.01a), b)	73.80 ± 6.00b)	91.40 ± 5.90a), b)	8.10 ± 0.10a), b)
Day	V_50_ + Lev			Metformin		
0	63.00 ± 5.60	56.80 ± 2.60	7.60 ± 0.03	59.40 ± 4.60	51.90 ± 3.40	7.30 ± 0.10
7	138.80 ± 8.30a)	154.50 ± 7.1a)	9.40 ± 0.10a)	160.00 ± 5.30a)	183.20 ± 3.00a)	9.60 ± 0.03a)
14	68.00 ± 4.10b)	56.50 ± 2.20b), c)	7.60 ± 0.03b), c)	65.80 ± 3.30b)	112.90 ± 2.90a), b)	8.20 ± 0.10a), b) ^*#^

Values are expressed as mean ± SEM. FPG = Fasting plasma blood glucose (mg dL^−1^); fTG = fasting triglycerides (mg mL^−1^); TyG = TyG Index = Ln[(triglycerides/fast blood glucose)/2]. Values are statistically significant at

a) *p* < 0.05 versus the control group;

b) *p* < 0.05 treated‐vanadium groups versus Dexa, and;

c) *p* < 0.05 V_50 _+ Lev group compared to the V_50_ group, using one–way ANOVA.

The [V^IV^O(H_2_O)(HEED)]_2_SO_4_ at 50 mg kg^−1^ (V_50_) was used to evaluate the biological effects of the vanadium complex alone, which exhibited reductions in fasting plasma glucose (FPG; ≈65%, *p* < 0.05) and fasting Triglycerides (fTG; ≈50%, *p* < 0.05), leading to a decrease in the TyG index (Triglyceride‐Glucose; 15%, *p* < 0.05) compared to the DEXA group at 14^th^ day of treatment.

As shown in Table 4, treatment with levan‐vanadium compounds at both doses (25 and 50 mg kg^−1^) resulted in ≈65% reductions in FPG and a dose‐dependent reduction in fTG and TyG index compared to the DEXA group at the end of treatment. Notably, the dexamethasone side effects on glucose metabolism were abolished by combining levan and the vanadium compound at the same dose (50 mg kg^−1^) (see Table 4). These findings suggest that including isolated levan as a vehicle can improve activity and reduce toxicity.^[^ ^60^ ^]^ Even at low concentrations, the composition is more effective in mitigating insulin resistance than the[V^IV^O(H_2_O)(HEED)]_2_SO_4_ complex alone.

The bacterial levan exhibits many well‐known properties, including biodegradability, self‐aggregation, encapsulation, controlled release capacity, water retention, immunomodulatory, antimicrobial, antidiabetic, and anticancer activity, as well as high biocompatibility and nontoxicity.^[^ ^61^ ^]^ These exceptional properties position levan as an attractive candidate for use as a nature‐based material in medicine, especially when combined with drugs to enhance its biological properties.

In this manuscript, we also present an alternative approach to designing a less toxic vanadium(V) compound for the treatment of diabetes and related metabolic disorders. Shang et al. recently reported that combination therapy with graphene quantum dots (GQDs) enhances efficacy while mitigating vanadium toxicity.^[^ ^62^ ^]^ The vanadium–GQDs combination reduces toxicity and enhances the antidiabetic effects, including improved control of hyperglycemia, increased insulin sensitivity, correction of hyperinsulinemia, and prevention of β‐cell loss. Similarly, other studies employed membrane‐permeable GQDs as a delivery vehicle for vanadium compounds.^[^ ^63^ ^,^ ^64^ ^]^ The GQDs enhanced the stability of the V compound, forming a complex that targets ligands and vanadium(V) for the selective regulation of Protein Tyrosine Phosphatase 1B (PTP1B) in both in vitro and in vivo settings.

In this study, levan was used as a delivery vehicle to enhance the antihyperglycemic effect of the vanadium complex in dexamethasone‐induced insulin‐resistant mice. Vanadium complexes, particularly the vanadyl(IV) ion could interact with levan, a β‐(2,6)‐linked fructan polysaccharide, primarily through coordination with the hydroxyl groups on the sugar units. Studies emphasizes that VO^2+^ exhibits a strong affinity for oxygen‐containing ligands, such as phosphates and carboxylates, and particularly for hydroxyl groups, which are abundant on levan. It is also noted that the speciation of vanadyl complexes is highly sensitive to the surrounding biological environment.^[^ ^65^ ^,^ ^66^ ^]^

Our results are consistent with previous studies,^[^ ^26^ ^,^ ^27^ ^]^ which demonstrated that the exopolysaccharide levan efficiently inhibits hyperglycemia and oxidative stress induced by diabetes. This suggests that levan supplementation in the diet may help prevent diabetic complications in adult rats. Kang et al. observed that supplementing the diet with up to 10% (m/m) levan decreased hyperlipidemia and triglyceride levels in obese rats.^[^ ^28^ ^]^ In agreement, Dahech et al. demonstrated that levan treatment reduced blood glucose levels by 52% in alloxan‐induced diabetic Wistar rats.^[^ ^26^ ^]^ Saeed et al. observed that using levan up to 5% (m/m) also significantly decreased total cholesterol and plasma glucose levels in diabetic rats.^[^ ^25^ ^]^

Furthermore, the strategy of using a new vanadium‐compound biocompatible formulation, employing an exopolysaccharide produced by *Zymomonas mobilis* as a vehicle, was based on the principle of "bait‐hook," which leads to an improvement in the drug's pharmacological properties.^[^ ^67^ ^]^ According to this principle, levan can be used as bait due to its biocompatibility, which enhances the biological effect of the vanadium compound in tissues such as the liver, adipose tissue, and skeletal muscle.^[^ ^66^ ^]^ In this context, the vanadium complex acts as the hook, and the composition enhances the treatment's overall efficacy.

A few studies have demonstrated that vanadium complexes can have enhanced stability, reduced toxicity, and improved biological activity compared to free vanadium compounds when administered in exopolysaccharide vehicles.^[^ ^68^, ^69^, ^70^ ^]^ For example, Woo et al. investigated the activity of the Bis(*N’*,*N’*‐dimethylbiguanide)oxidovanadium(IV) complex (VO(metf)_2_), using the drug metformin as a ligand, administered in a matrix of Arabic gum 3% (m/m), showing 50% glucose plasmatic levels decreasing in STZ‐induced diabetic rats.^[^ ^24^ ^]^ Additionally, Yuen et al. demonstrated that Zucker Diabetic Fatty (ZDF) rats treated with the Bis(maltolato)oxidovanadium(IV) (BMOV) complex and 3% Arabic gum (m/m) (a polysaccharide) exhibited reduced triglyceride levels and hyperglycemia, while also preserving pancreatic β‐cell function.^[^ ^71^ ^]^

The TyG index is a screening parameter for insulin resistance, calculated as the ratio of triglyceride levels to plasma glucose levels, and is utilized to estimate peripheral insulin sensitivity.^[^ ^39^ ^]^ In our study, all groups received a vanadium compound, and the formulations containing vanadium and levan (V + Levan) at both doses (25 and 50 mg kg^−1^) demonstrated a reduction in the TyG index by the 14th day of dexamethasone treatment. Our results indicate that the novel vanadium complex and both formulations containing the exopolysaccharide levan, even at low concentrations, exhibit significant antihyperglycemic effects and attenuate insulin resistance induced by synthetic glucocorticoid treatment. The controlled‐release properties of polysaccharide matrices enable sustained delivery, which is advantageous for long‐term therapeutic applications. ^[^ ^72^, ^73^, ^74^ ^]^

## 4 Conclusion

This study demonstrates that incorporating isolated levan as a vehicle for the oxidovanadium(IV) complex with *N*‐(2‐hydroxyethyl)ethylenediaminoate enhances its activity and reduces toxicity, even at low concentrations. This bioactive composition is more effective in mitigating glucocorticoid‐induced insulin resistance in mice than the [V^IV^O(H_2_O)(HEED)]_2_SO_4_ complex alone.

The acute oral toxicity test ranked the novel vanadium complex toxicity in category 4 (estimated LD_50_ between 300 and 2,000 mg kg^−1^), supporting its suitability for biomedical applications. The biochemical and hematological parameters showed no significant differences between the vanadium‐treated and control groups, indicating that the compound did not exhibit nephrotoxicity or hepatotoxicity at the tested doses.

However, a significant concern is that the TyG index reflects the extent of insulin resistance, and the status of key pancreatic function‐related factors, such as basal insulin levels and insulin secretion in response to glucose stimulation, could be further investigated in future experiments.

In conclusion, the combination of the vanadium compound with levan exopolysaccharide is safe and enhances insulin action in a mouse model of dexamethasone‐induced insulin resistance. Furthermore, the levan bioconjugate with a novel vanadium–amino alcohol complex represents a promising approach for treating insulin resistance. These findings support the potential of levan as a bioactive carrier to improve the pharmacological properties of new antidiabetic agents.

## Conflict of Interest

The authors declare no conflict of interest.

## Supporting information

Supplementary Material

## Data Availability

Research data are not shared.
