# Supplementary material for: Evaluation of the Therapeutic Potential of Bioactive Materials Based on a Complex of Oxidovanadium(IV) and Exopolysaccharide Levan in a Model of Insulin Resistance in Mice
Source: ChemMedChem. 2025 Dec 17;21(4):e202500754. doi: 10.1002/cmdc.202500754 (PMC12913243; doi:10.1002/cmdc.202500754)

SUPPORTING INFORMATION

Figure 1S. NMR ^1H (300 MHz) of Levan, in D_2O.

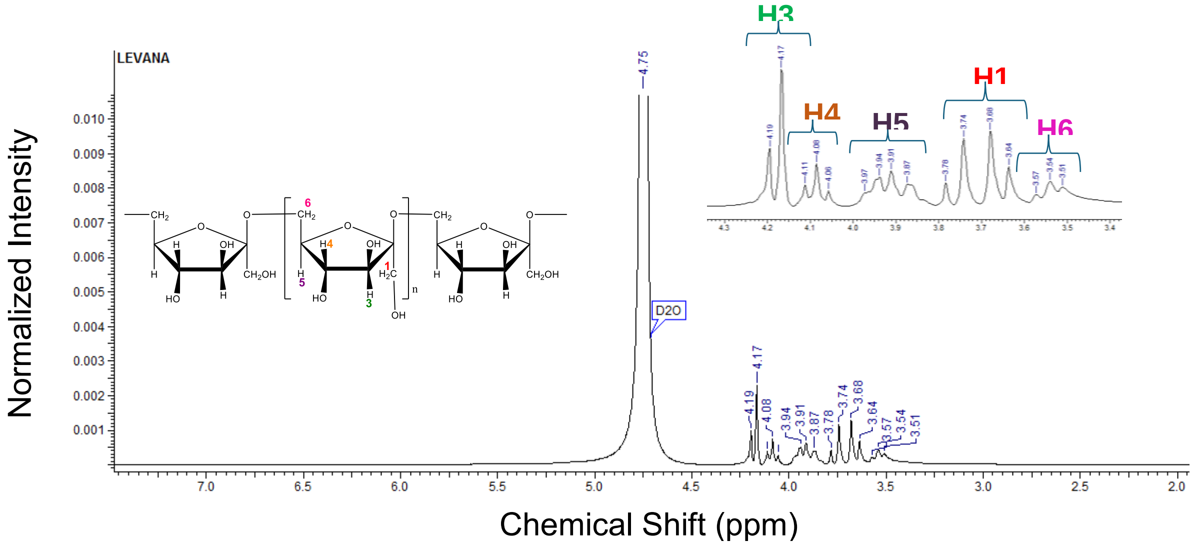

Figure 2S. NMR ^{13}C (100 MHz) of Levan, in D_2O.

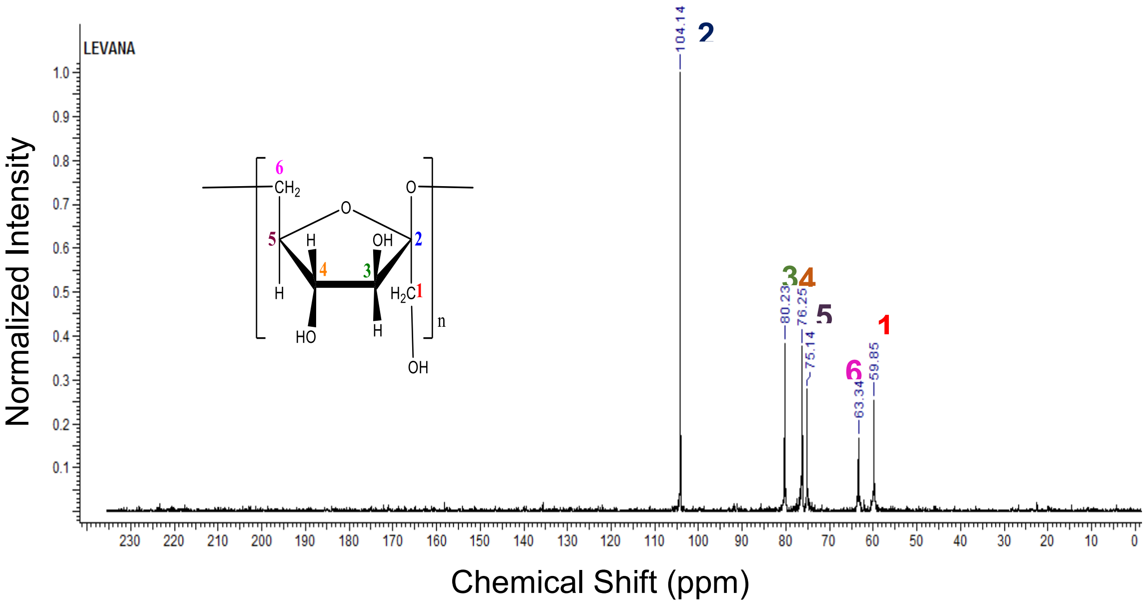

Figure 3S. NMR ^{1}H (300 MHz) of $[V^{IV}(HEED)(H_2O)].3H_2O$, in D_2O.

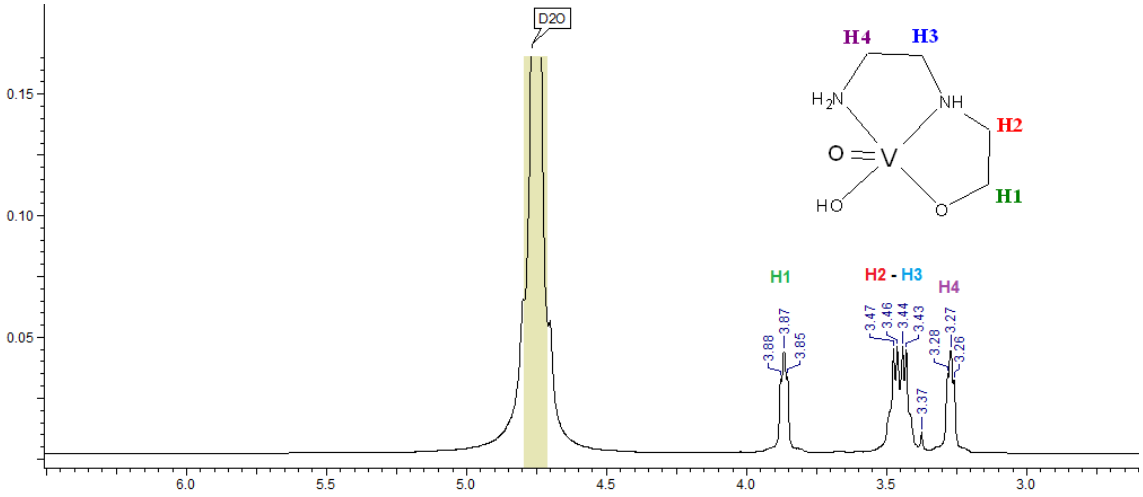

Figure 4S. NMR ^{13}C (100 MHz) of $[V^{IV}(HEED)(H_2O)].3H_2O$, in D_2O.

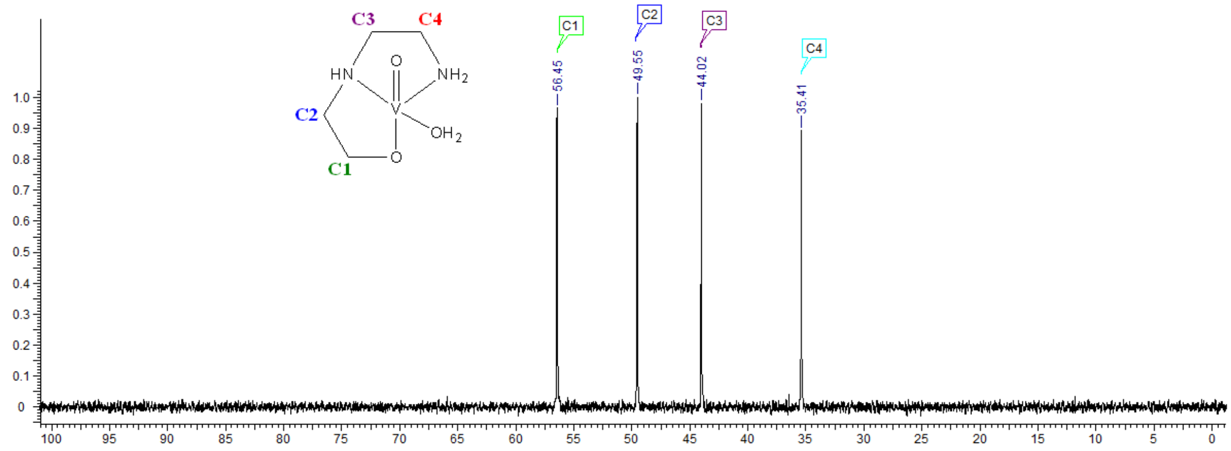

Figure 5S. FTIR spectrum of the HEED (free ligand) (a), $VOSO_4$ (b) and $[V^{IV}(HEED)(H_2O)].3H_2O$ (c) complex in ATR.

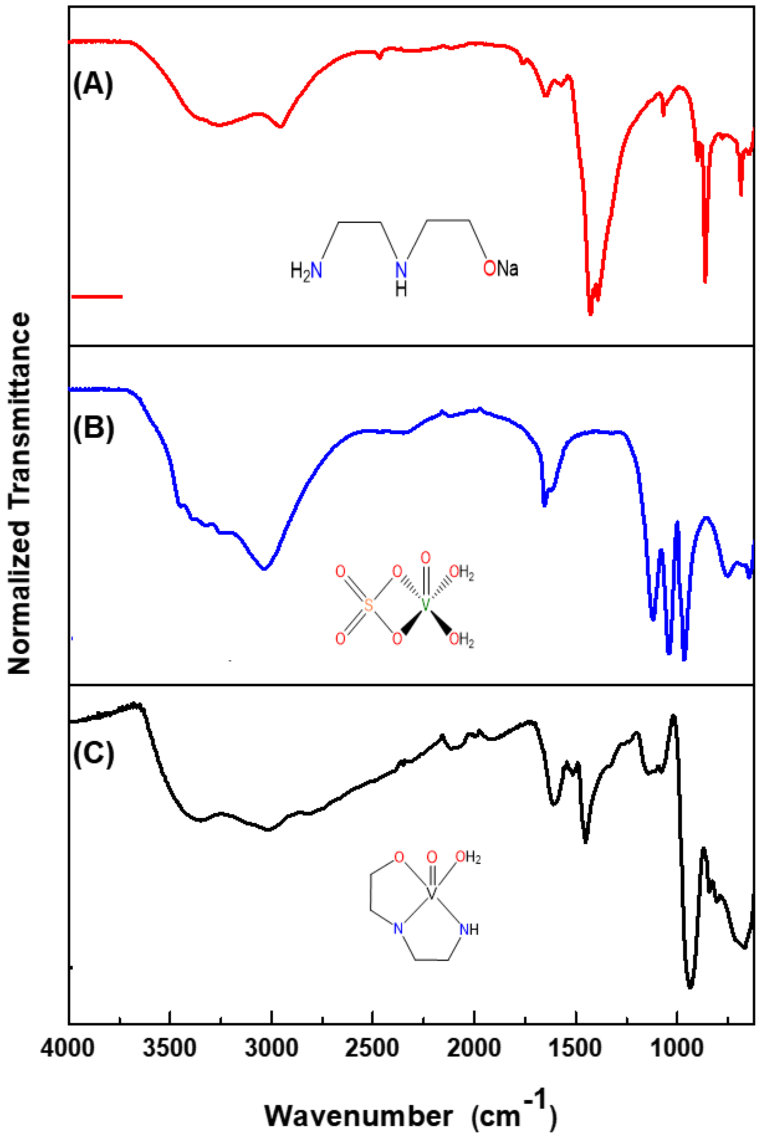

Supplement: Supplementary file 1 — Supplementary Material [file CMDC-21-e202500754-s001.pdf]
